# Intensive production of the harpacticoid copepod *Tigriopus californicus* in a zero-effluent ‘green water’ bioreactor

**DOI:** 10.1038/s41598-021-04516-w

**Published:** 2022-01-10

**Authors:** Alfonso Prado-Cabrero, Rafael Herena-Garcia, John M. Nolan

**Affiliations:** 1 Nutrition Research Center Ireland, School of Health Science, Carriganore House, Waterford Institute of Technology, West Campus, Carriganore, X91K236 Waterford Ireland; 2 Blueming SL, Murcia, Spain

**Keywords:** Industrial microbiology, Marine biology

## Abstract

Aquaculture is looking for substitutes for fishmeal and fish oil to maintain its continued growth. Zooplankton is the most nutritious option, but its controlled mass production has not yet been achieved. In this context, we have developed a monoalgal ‘green water’ closed-loop bioreactor with the microalgae *Tetraselmis chui* that continuously produced the harpacticoid copepod *Tigriopus californicus*. During 145 days of operation, the 2.2 m^3^ bioreactor produced 3.9 kg (wet weight) of *Tigriopus* with (dry weight) 0.79 ± 0.29% eicosapentaenoic acid (EPA), 0.82 ± 0.26% docosahexaenoic acid (DHA), 1.89 ± 0,60% 3S,3’S-astaxanthin and an essential amino acid index (EAAI) of 97% for juvenile Atlantic salmon. The reactor kept the pH stable over the operation time (pH 8.81 ± 0.40 in the algae phase and pH 8.22 ± 2.96 in the zooplankton phase), while constantly removed nitrate (322.6 mg L^−1^) and phosphate (20.4 mg L^−1^) from the water. As a result of the stable pH and nutrient removal, the bioreactor achieved zero effluent discharges. The upscaling of monoalgal, closed-loop ‘green water’ bioreactors could help standardize zooplankton mass production to supply the aquafeeds industry.

## Introduction

As a consequence of a fast growth over the last 20 years, fed-aquaculture has recently outpaced non-fed aquaculture production of aquatic animals^1^. This trend, together with the stagnation of forage fish captures since the 1980s^2^ is stressing the production of nutritious and cost-effective aquafeeds^3^. The uncertainty about the availability of forage fish, the consequent rising prices of this commodity and the growing public awareness of the ecological impact of this fishing activity^4^ are driving producers to find environmentally friendly nutrients to substitute this raw material^5,6^. Hence, since the 1990s, industry and researchers have been working to replace fishmeal and fish oil with plant meal and fish and animal by-products. Today, these materials are commonly found in formulations available on the market^7^. Due to the limited capacity of these substitutes, new raw materials such as insect larvae^8^, genetically modified crops^9^, macroalgae^10^, microalgae^11^, yeast^12^, bacteria^13^, and bioflocs^14^ are being studied to join the list of alternatives to forage fish. Nevertheless, these new raw materials also face challenges, such as their limited availability^5^, their environmental impact^15^ or the potential public reluctance to their use^16^.

Zooplankton is the most nutritious feed for fish, but nowadays, its use to replace fish meal and fish oil is unthinkable. Although *Artemia* eggs obtained from natural ponds, copepod eggs produced by various companies and rotifers produced on-site are used in hatcheries^17^, mass and controlled production of zooplankton has not yet been achieved. Attempts at small or medium scale have been reported, such as with the harpacticoid copepod *Tigriopus japonicus* and the calanoid copepod *Acartia tonsa* in batch culture^18,19^, with *Artemia* in a flow-through system^20^, or with the harpacticoid copepod *Amphiascoides atopus* in a recirculated system^21^. Nevertheless, production scales achieved or continuity are still insufficient to help replace forage fish. Technical difficulties such as the production and supply of cheap and quality feed, maintenance of water quality and prevention of contamination by invasive species^22^ have not been yet solved. Zooplankton mass production must integrate these requirements into a system that minimizes the need for human labor, is largely automated, is unaffected by weather conditions and is environmentally friendly^22^.

In 2018, ‘green water’ aquaculture was responsible for 32% of non-fed aquaculture production or 10% of global aquaculture production^1^. Aquaculturists in Southeast Asia have used this technique for millennia to produce herbivorous fish such as tilapia and carp at meager costs, since the feed for these fish species is microalgae and bacteria that grow in the ponds when aquaculturists fertilize the water^23^. Thus, ‘green water’ aquaculture produces fish feed on site, but in addition, the rich biodiversity of these ecosystems protect farmed fish and shrimps against diseases, and maintains water quality in a way that minimizes the production of contaminated effluents^11^. Importantly, protozoa and zooplankton are common inhabitants of these ecosystems, thus making the ‘green water’ technique interesting for studying controlled zooplankton production.

*Tetraselmis* is a marine microalgae widely used in aquaculture for its high nutritional value^24^. Recently, the large-scale production of this genus has been reported in industrial photobioreactors^25^, and its bioremediation capabilities, both in aquaculture^26^ and in domestic wastewater^27^ are very promising. Furthermore, *Tetraselmis* can grow in reused culture media^28^ or even benefit from them^29^. These characteristics make *Tetraselmis* an ideal candidate to inoculate a recirculated ‘green water’ culture to feed zooplankton and control the levels of ammonium and phosphate in the water. The harpacticoid copepod *Tigriopus* has already been tested for mass production^18^, and it has been used successfully to feed sea bream^30–32^. *Tigriopus* has an epi-benthic behaviour, feeds on decaying matter and produces the valuable omega-3 fatty acid DHA (docosahexaenoic acid) *de n*ovo^33^ and the carotenoid astaxanthin from ingested carotenoids^34,35^. Naturally inhabiting rocky tidal pools, *Tigriopus* has a great capacity to deal with wide ranges of temperature and salinity in the water^36^. Due to these capabilities, *Tigriopus* is an excellent candidate for mass production.

The present work describes the use of *Tetraselmis chui* in a monoalgal ‘green water’ closed-loop bioreactor to continuously produce the harpacticoid copepod *Tigriopus californicus*. The bioreactor was fed only with sunlight, atmospheric air, nitrate, phosphate, metals and vitamins. In addition to serving as feed, *Tetraselmis* and the other resident microorganisms contributed to maintaining water quality, allowing the achievement of zero effluent discharges. We collected significant amounts of *Tigriopus* rich in essential amino acids, astaxanthin, EPA and DHA. To the authors' knowledge, this is the first report describing the use of the ‘green water’ technique to produce zooplankton in a controlled way. This new concept has the potential to be scaled up to help substitute forage fish and contribute to the sustainable growth of aquaculture.

## Results

### Reactor construction

**Figure 1:**
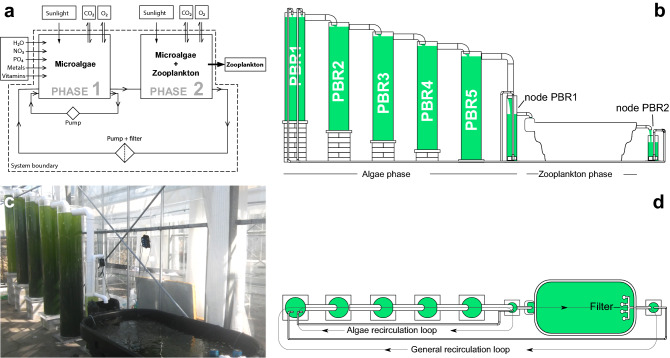
Scheme and view of the self-sustainable ‘green water’ polyculture bioreactor. (**a**) Scheme of the bioreactor, with inputs and outputs. (**b**) Side view of the bioreactor showing key parts. (**c**) Picture of the bioreactor (**d**) top view of the bioreactor showing the position of the filter and water flow directions.

**Figure 2:**
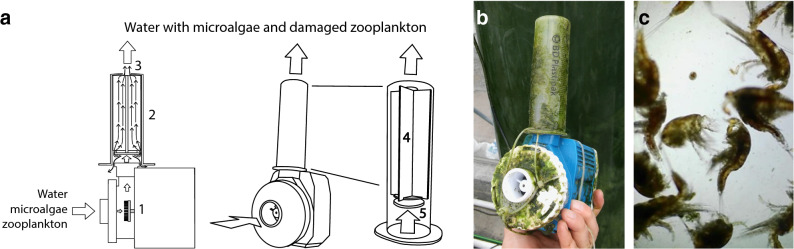
Anti-grazer device. (**a**) Scheme of the anti-grazer device showing the water entrance and exit, and a magnification of the buffer surface and water exit*.* (1) Needle wheel impeller; (2) semi-closed scape cylinder; (3) scape hole; (4) plunger; (5) plunger flange (**b**) Picture of one of the anti-grazer devices that worked in one of the photobioreactors. (**c**) Appearance of *Tigriopus californicus* after using an anti-grazer device for 10 min in a container with 15 L of water from the bioreactor.

The bioreactor was built and trialled in the glasshouse located at Carriganore House (Waterford, Ireland). The algae phase consisted of five photobioreactors (PBR) with a total water capacity of 1200 L. The five PBRs were made up of clear acrylic cylinders of 400 mm outer diameter (o/d), 8 mm thickness and 2000 mm height. The PBRs were placed at increasing heights (Fig. 1b, c) and were interconnected by UPVC pipes to allow water flow among them by overflow. Aeration was provided to the PBRs with an air pump through airlines. A smaller PBR of 200 mm o/d, 5 mm thickness and 1300 mm height (hereinafter referred to as node PBR1) was constructed to receive water from PBR5, located at the end of the cascade (Fig. 1b, d). Node PBR1 held a water pump to pump water back to PBR1 at the top of the cascade, thus enabling a water recirculation loop among the algae phase (algae recirculation loop, Fig. 1d) at circa 30 L/min. This recirculation loop was intended to promote culture media mixing and gas exchange with the atmosphere within this phase. UPVC pipes were joined using PVC pipe cement*.* To facilitate bacteria proliferation within the bioreactor, three bags (1.2 Kg in total) of filtering media (ceramic rings) were deposited in node PBR1. This node also equipped an overflow pipe to allow the flow of water to the zooplankton phase. Each PBR was equipped with an anti-grazer device (Fig. 2) to prevent the growth of zooplankton (both internal and foreign) in the microalgae phase. The bioreactor occupied an area of approximately 16 m^2^, including the surrounding space for the movement of personnel.

The zooplankton phase consisted of a water trough of 1000 L of capacity (Fig. 1), with a dispositive to automatically replenish tap water when water levels decreased due to evaporation. Air was inoculated in this phase using an air pump into three sponge filters placed at the bottom of the tank. Water was heated using aquarium heaters. An in-house built 75 µm mechanical filter filtered out zooplankton from water exiting the tank to a second node PBR of 200 mm o/d, 5 mm thickness and 500 mm height (node PBR2 in Fig. 1b). Inside node PBR2, another water pump pumped the filtered water back to PBR1 at the top of the cascade, thus constituting the general recirculation loop (Fig. 1d). Flow among algae and zooplankton phases was dictated by the efficiency of the filter in the zooplankton phase, yielding a water flow of 1–2 L/min in average.

### Anti-grazer device

**Figure 3:**
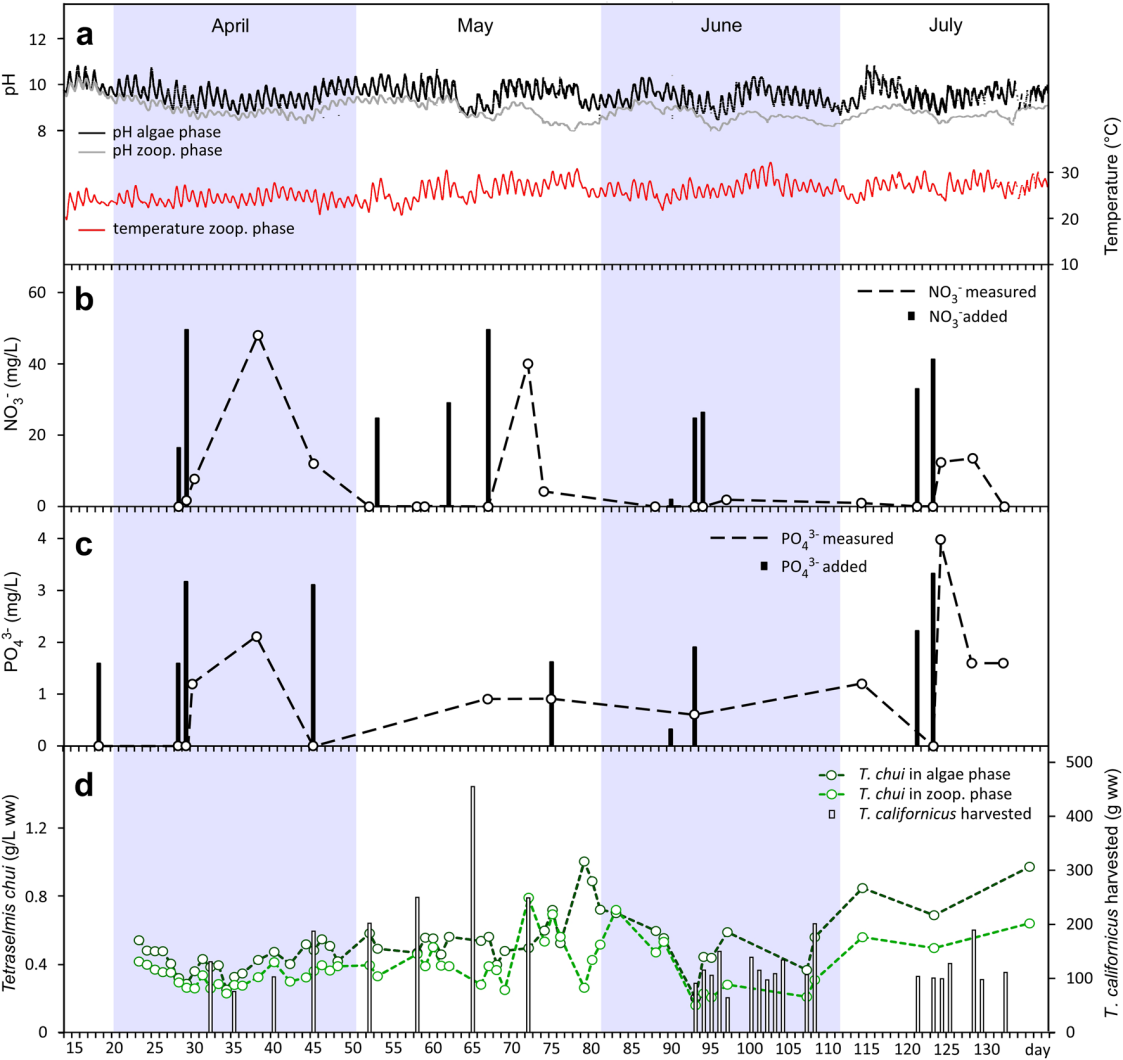
Bioreactor monitoring and harvesting. (**a**) pH and temperature evolution. The black and the upper grey line represent pH evolution in the algae and zooplankton phases, respectively. The lower red line stands for temperature evolution in the zooplankton phase. (**b**) Nitrate concentration and nitrate additions. The black dashed line stands for the evolution of the nitrate concentrations in the zooplankton phase. The black bars stand for nitrate added to the bioreactor. (**c**) Phosphate concentrations and phosphate additions. The black dashed line stands for the evolution of phosphate concentrations in the zooplankton phase. The black bars stand for the amounts of phosphate added to the bioreactor. (**d**) Microalgae evolution and *T. californicus* harvesting events. The dashed dark green line represents microalgae density in the algae phase, and the light green dashed line represents the algae density in the zooplankton phase. The bars stand for *T. californicus* biomass harvesting events, showing the amount harvested in each case.

**Figure 4:**
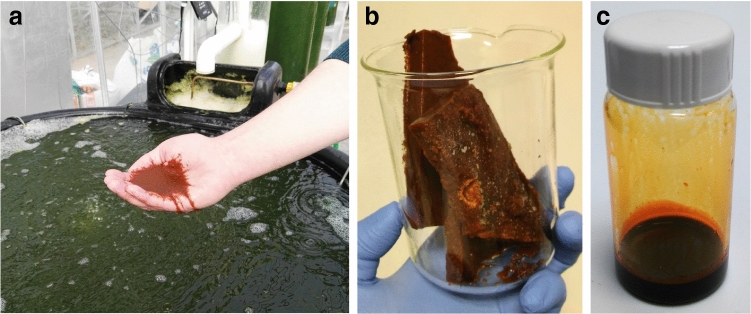
*Tigriopus californicus* in the bioreactor, frozen biomass and extracted oil. (**a**) *T. californicus* as harvested by netting. In the background, the zooplankton phase of the bioreactor with reddish coloured water due to the presence of this crustacean. Behind the zooplankton phase, the green coloured photobioreactors due to the microalgae *Tetraselmis chui*. (**b**) Frozen *T. californicus* biomass. (**c**) Oil extracted from *T. californicus* biomass.

**Figure 5:**
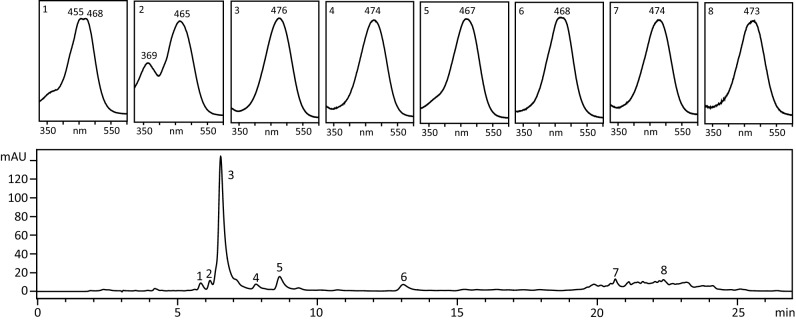
High-performance liquid chromatography (HPLC) chromatogram of the carotenoids present in oil from *Tigriopus californicus*. Each peak in the chromatogram (lower panel) is numbered, and its spectrum is shown in the upper panel, with the maximum absorbance. Peak 1, unknown; peak 2, astaxanthin cis-isomer; peak 3, all-trans 3S,3’S-astaxanthin, peak 4, unknown; peak 5, unknown; peak 7, 3-hydroxyechinenone (tentative); peaks 7 and 8, astaxanthin esters.

The filter built and installed in the bioreactor to prevent zooplankton migration to the algae phase occasionally failed. As a result, *Tigriopus* individuals migrated to the algae phase and settled at the bottom of the PBRs. To prevent extensive colonization of the PBRs, we designed a device to destroy filter escaping *Tigriopus* (Fig. 2). The anti-grazer device's base consisted of an aquarium skimmer pump (Bubble Magus DSP4000), equipped with a needle wheel impeller (Fig. 2a, 1) that inflicts damage on the absorbed zooplankton. Using the parts of a 100 ml plastic needle, we constructed an internally semi-capped cylinder to absorb the pump's outflow and inflict additional damage to the zooplankton. The barrel of the syringe (Fig. 2a, 2), with a 1 cm escape hole drilled at its end (Fig. 2a, 3), constituted the water exit. The plunger seal was discarded, and the plunger was inserted in the barrel in reverse (Fig. 2a, 4), converting the plunger into a cap occupying 80% of the internal diameter of the barrel (Fig. 2a, 5). This modified syringe was loosely attached perpendicularly to the outlet of the pump using cable ties (Fig. 2b). Thus, the zooplankton entering the pump got damaged by the impeller, and after exiting the pump, it suffered further damage by colliding with the plunger (Fig. 2c). Finally, the damaged zooplankton bounced off the cylinder or exited through the hole in its end. We built five units of this anti-grazer device and on day 65 we finished installing one of them at the bottom of each of the five PBRs. In addition to preventing the invasion of zooplankton in the algae phase, the anti-grazer devices increased the water mix in this phase and contributed to keeping the bottom of the PBRs clear of decaying matter.

### Bioreactor trial

The bioreactor was inoculated on March 13th, 2017, with 30 L of *Tetraselmis chui* (0.45 g L^−1^ ww). On March 18th, 26 g (ww) of *Tigriopus californicus* cultured in two 10 L aquariums were added. March 18th was set as the starting point of the experiment. Gradually, the bioreactor acquired different populations of bacteria and fungi from the environment. The identification of these opportunistic microorganisms is outside the scope of this work. The trial was ended on August 8th, 2017, completing a running time of 145 days.

### pH and temperature

The pH was recorded continuously in the algae and zooplankton phases. In the algae phase (upper black line in Fig. 3a), the pH followed a daily cycle with the minimum at midnight and the maximum in the afternoon, coinciding with the period of maximum insolation. Despite the daily fluctuation, the pH remained stable in this phase during the bioreactor's operation at 8.81 ± 0.40 (SD). The pH in the zooplankton phase (grey line in Fig. 3a) followed a similar pattern, although with less pronounced intra-day fluctuations, higher inter-day fluctuations and lower average value (8.22 ± 2.96).

The temperature was monitored in the zooplankton phase (lower red line in Fig. 3a). This parameter, like the pH, showed a daily cycle, with the highest temperatures generally reached in the afternoon. The average temperature was 25.59 ± 4.60 °C.

### Nitrogen, phosphorus, trace elements and vitamins

The nitrate and phosphate levels in the bioreactor were monitored with punctual measurements of water collected from PBR5 from the algae phase. Fig. 3b shows that nitrate levels (black dashed line) decreased to zero in the first 30 days of operation. To maintain the growth of the microalgae, we made new sodium nitrate additions. Still, this decreasing trend in nitrate concentration was constant throughout the trial, despite the continuous sodium nitrate additions we performed. In total, 322.62 mg L^−1^ of nitrate (in the form of sodium nitrate) was added to the bioreactor during the trial, or 66.75 mg L^−1^ month^−1^, and its totality was removed by the ecosystem resident in the bioreactor. Fig. 3c shows that the trend of phosphate (black dashed line) was similar to that of nitrate. A total of 20.43 mg L^−1^ of phosphate (black bars) was added during the trial, or 4.23 mg L^−1^ month^−1^. The resulting ratio of nitrate:phosphate added during the running period was 15.8:1, very similar to that used to set the trial (15.7:1).

We monitored iron in the bioreactor as a proxy for trace metal levels. We measured iron concentrations periodically, and when its levels approached zero, we added the necessary amount of trace elements stock solution (as defined in the F/2 medium) to the bioreactor. Thus, in total, we added trace elements containing 0.47 mg L^−1^ of iron, or 0.10 mg L^−1^ month^−1^ of iron at days 29, 53 and 90.

On day 42, we added to the bioreactor 400 mg of Vitamin B1, 2 mg of Vit H and 2 mg of Vit B12, and on day 71, we added 240 mg of Vitamin B1, 1.2 mg of Vit H and 1.2 mg of Vit B12.

### Microalgae

Microalgae was monitored in both phases of the bioreactor. Dark green border circles and light green border circles in Fig. 3d represent microalgae concentrations (g/L ww) in the algae and zooplankton phases, respectively. As this Figure shows, microalgae concentrations were generally lower in the zooplankton phase (0.038 ± 0.14 g/L ww) than in the algae phase (0.52 ± 0.16 g/L ww).

### Zooplankton productivity

Bars in Fig. 3d represent zooplankton harvesting events (g wet weight). From mid-April to the end of May, we harvested roughly every 5 days (Table 1, days 32–72), with an average yield of 207 ± 119 g (ww) per harvest*.* In June and July (Table 1, days 93–108 and 121–132), we carried out two periods of daily collections, which involved capturing an average of 121 ± 40 g (ww) of zooplankton and 129 ± 42 g (ww), respectively. In total, during the entire operating period, we collected *Tigriopus californicus* on 27 different days, producing a total of 3.92 Kg of biomass (ww). This figure can be projected to a productivity of 9.86 Kg bioreactor^−1^ year^−1^ or 0.62 Kg m^−2^ year^−1^) (Table 1). The average dry weight of the zooplankton was 14.96 ± 1.90 % (*n* = 27).

**Table 1:** Productivity of *Tigriopus californicus* in the bioreactor at the different harvesting rates (wet weight).

Days	Harvesting events	Harvesting rate (days)	Harvested (Kg total)	Harvested (g per harvest)	Productivity (Kg reactor^-1^ year^-1^)	Productivity (Kg m^-2^ year^-1^)*
32–72	1–8	5.13	1.654	207 ± 119	15.09	0.920
93–108	9–20	1.25	1.343	121 ± 40	32.68	2.042
121–132	21–27	1.57	0.831	129 ± 42	27.56	1.723
Total	27	5.37	3.917	145 ± 79	9.86	0.616

*The area occupied by the bioreactor was 16 m^2^.

### Zooplankton biomass characterization

We characterized *Tigriopus* biomass (Fig. 4a, b) in three biomasses collected during the second harvest period (Table 1, days 93–108, harvests 10, 14 and 19). The composition of *Tigriopus* biomass (harvesting number 10) is represented in Table 2. The dry weight of the three biomasses was on average 13.9 ± 0.3 % of wet weight (measured in-house), which agrees with moisture content measured externally (84.6 % moisture, see Material and methods).

**Table 2:** *Tigriopus californicus* biomass proximate composition (g/100 g dry weight).

Proximate analysis		Essential amino acids		Non-essential amino acids	
Moisture	84.6	Histidine	1.36	Alanine	3.70
Protein	66.0	Iso-leucine	2.73	Aspartic acid	4.16
Total fat	9.7	Leucine	3.31	Glutamic acid	4.16
Ash	8.4	Lysine	3.57	Glycine	2.99
Total carbohydrate	16.2	Methionine	1.17	Proline	4.35
Total dietary fibre	6.5	Phenylalanine	2.73	Serine	2.73
Available carbohydrate	9.7	Threonine	2.79	Tyrosine	2.79
Total sugar	2.6	Valine	3.05	Cystine	1.23
Sodium	1.5	Arginine	5.97		
Gross energy	261				

Moisture as percentage of loss on drying. Gross energy as kJ/100 g ww.

We also measured the protein content of *Tigriopus* in-house, as it was estimated externally using the nitrogen-to-protein Jone’s conversion factor^37^ at 66% of dry weight (Table 2), and this method is generally considered inaccurate^38^. The BCA method (see Material and methods) yielded a protein content of 35.7 ± 3.5% of dry weight*.*

We evaluated the composition of essential amino acids of *Tigriopus* biomass (Table 2) calculating the essential amino acids index (EAAI)^39^ for juvenile Atlantic salmon (*Salmo salar*) as example^40^. This index compares the composition of essential amino acids in a feeding material with the composition of these essential amino acids in a feeding material of proven efficacy for a particular species at a particular life stage. For our estimation, as there is no apparent amino acid digestibility coefficient published for *Tigriopus*, we adopted the lower range of this coefficient calculated for the calanoid copepod *Calanus finmarchicus*^41^, which was 91%. As a result, the EAAI estimated for juvenile Atlantic salmon of *Tigriopus* biomass was 97%.

### Oil composition

We studied the fat-soluble fraction of *Tigriopus* on the extracted oils (Fig. 4c). The dry biomasses of harvests 10, 14 and 19 contained 13.1 ± 4.2 % of oil. The average astaxanthin content in the oils, estimated by spectrophotometry, was 14.5 ± 2.0 mg g^−1^ of oil. The HPLC chromatogram shown in Fig. 5 represents the carotenoids isolated from harvest 14 as a representative example. The main peak in this Figure (peak 3), shows the retention time and spectrum of free astaxanthin. The stereoisomeric configuration of astaxanthin in *T. californicus* (3S,3’S) was recently described by our group^35^. This peak accounts for 85.9 ± 0.9 % of total carotenoids in the chromatogram. The rest of the peaks were not identified (peaks 1, 4 and 5), identified as an astaxanthin cis isomer (peak 2), previously tentatively identified as 3-hydroxyechinenone^35^ (peak 6) or astaxanthin esters (peaks 7 and 8).

We analysed the fatty acid profile of *Tigriopus* oil (TO), and to put the results into context, we analysed in parallel commercial krill oil (KO) and Calanus oil (CO), both rich in omega-3 fatty acids. Table 3 shows the average fatty acid composition of the three oils extracted from *Tigriopus*, and the average fatty acid composition of Prowise SUPERBA*krill* Oil (*n* = 2) and Arctic Ruby (Calanus) oil (*n* = 2). The content of saturated fatty acids in TO was different from that of KO and CO: Myristic acid was significantly lower than in the other two oils (KO, *p* = 0.041; CO, *p* = 0.038), and palmitic acid was similar to that of KO and significantly lower than CO (*p* = 0.002). Stearic acid, however, was significantly higher in TO than in the other two oils (KO, *p* = 0.008; CO, *p* = 0.006). The content of fatty acids with two unsaturations (linoleic acid) or three unsaturations (the omega-6 γ-linolenic acid and the omega-3 α-linolenic acid) was significantly higher in TO (*p* = 0.000 and *p* = 0.000 for linoleic acid, *p* = 0.002 and *p* = 0.003 for γ-linolenic acid and *p* = 0.000 and *p* = 0.001 for α-linolenic acid). Stearidonic acid, an omega-3 with four unsaturations, was similar in all three oils. The omega-3 EPA was significantly lower in TO with respect to the other two oils (KO, *p* = 0.030; CO; *p* = 0.017), and the omega-3 DHA was statistically similar in the three oils. In the fatty acid sums, the total of saturated fatty acids in TO was significantly lower than in KO (*p* = 0.032), and the total of omega-3 in TO was significantly higher than that of KO.

**Table 3:** Fatty acid composition of oil extracted from *Tigriopus californicus*, and fatty acid composition of commercial oils from *Euphasia superba* (Prowise SUPERBA*Krill*™ Oil) and *Calanus finmarchicus* (Arctic Ruby® Oil).

Fatty acid	*Tigriopus californicus*	*Euphasia superba*	*Calanus finmarchicus*
C14:0 (miristic acid)	3.6 ± 0.1^ab^	140.0 ± 12.4	106.8 ± 8.7
C16:0 (palmitic acid)	160.2 ± 10.0^b^	188.1 ± 21.0	73.2 ± 6.5
C16:1 (palmitoleic acid)	8.7 ± 0.7^ab^	81.0 ± 7.4	26.6 ± 2.1
C18:0 (stearic acid)	81.0 ± 13.9^ab^	14.0 ± 2.2	10.2 ± 1.5
C18:1n-9c (oleic acid)	140.9 ± 23.9^b^	129.6 ± 12.7	32.4 ± 6.5
C18:1n-11c (vaccenic acid)	41.4 ± 7.2^a^	80.1 ± 6.1	15.8 ± 15.4
C18:2n-6 (linoleic acid)	67.6 ± 4.1^ab^	15.7 ± 1.4	7.2 ± 0.5
C18:3n-6 (γ-linolenic acid)	8.5 ± 0.9^ab^	2.2 ± 0.2	0.8 ± 1.1
C18:3n-3 (α-linolenic acid)	106.2 ± 7.3^ab^	10.9 ± 0.9	20.3 ± 1.7
C18:4n-3 (stearidonic acid)	15.3 ± 0.9	46.2 ± 8.1	73.7 ± 10.9
C20:5n-3 (EPA)	59.8 ± 9.6^ab^	93.9 ± 9.6	100.7 ± 8.4
C22:5n-3 (DPA)	12.1 ± 1.6^ab^	2.1 ± 0.3	3.3 ± 0.3
C22:6n-3 (DHA)	63.2 ± 12.1	43.8 ± 4.3	63.3 ± 5.9
Total SFA	244.8 ± 23.6^a^	342.1 ± 35.5	190.3 ± 16.7
Total Omega-3	256.6 ± 14.7^a^	196.8 ± 23.3	261.4 ± 5.4
Total fatty acids	768.6 ± 57.9	847.4 ± 86.5	534.4 ± 47.8

Units as mg of free fatty acids per g of oil, mean ± SD. ^a^Significantly different between *Tigriopus* and *Euphasia superba*; ^b^significantly different between *Tigriopus* and *Calanus*. Total Saturated Fatty Acids (SFA): sum of C14:0, C16:0 and C18:0. Total Omega-3: sum of C18:3n-3, C18:4n-4, C20:5n-3 and C22:6n-3.

## Discussion

We have developed a monoalgal ‘green water’ closed-loop bioreactor that has produced *Tigriopus californicus* on a pilot scale for more than three months. The resident microbial ecosystem fed the zooplankton and facilitated the maintenance of water quality. To carry out the latter, the resident microbial ecosystem constantly reduced the levels of nitrogen, phosphorus and metals in the water, even in the presence of the fecal pellets of *Tigriopus*^43^. Although we cannot conclude with the work described here, we hypothesize that the high capacity of *Tetraselmis* to remove nitrogen from water^26,27^ was essential in this task. Thus, we simply had to periodically add nitrate, phosphate, metals and vitamins to keep the microalgae fit. The stable pH resulted from the activity of the microbial ecosystem plus the zooplankton, which contributed with the carbonic dioxide of its respiration. Due to the stable pH and nutrient deficit, we achieved zero effluent discharges, thus apparently surpassing the low effluent discharges characteristic of 'green water' aquaculture. In this recirculated medium, *Tigriopus* bloomed constantly, and *Tetraselmis* achieved one of the longest microalgae cultures reported to date^42^. Furthermore, and echoing the resilience of 'green water' aquaculture to contamination, the continued appearance of dead insects and occasionally birds in the zooplankton phase did not affect either the microalgae or the zooplankton.

The water flow rate in the bioreactor allowed each phase to keep distinctive characteristics. The intraday pH fluctuation was lower in the zooplankton phase, probably due to a lower degree of photosynthesis in this phase. The average pH in this phase was also lower, likely due to the carbonic acid expelled by *Tigriopus*. Finally, the density of *Tetraselmis chui* in the zooplankton phase was, again, lower. This could be possibly due to the tendency of *Tetraselmis* to precipitate^44^, as a less extensive water agitation was performed in this phase. These precipitated microalgae constituted the feed for *Tigriopus*, which, as an epi-benthic crustacean, feeds exclusively on detritus^45^.

To compare the productivity of our bioreactor with the most significant attempt made to date of mass cultivation of zooplankton^18^, we omitted our microalgae phase, since in this trial, carried out by Fukusho, yeast was used as food, whose production was not described. Thus, taking their highest productivity (168 kg in 210 m^3^ and 89 days), their projected productivity was 3.28 kg m^−3^ year^−1^. The productivity of our bioreactor was 3.9 kg in 1 m^3^ and 145 days, which projects to 9.86 kg m^−3^ year^−1^, which is three times higher. Of the two harvesting rates we tested over the trial, the daily harvest appeared to perform better than the weekly harvest in terms of productivity. However, we cannot attribute this higher performance to the harvesting rate, as the bioreactor conditions during those days could have been more favourable (the bioreactor run inside a glasshouse, subject to weather conditions). Nevertheless, research suggests that more frequent harvests help keep a lower zooplankton density in the tank, thus favouring *Tigriopus* reproduction and nauplii development^46^.

*Tigriopus* biomass had a remarkable composition. The essential amino acids and the DHA it contained could potentially meet the nutritional needs of juvenile salmon. Likewise, its high astaxanthin concentrations could help replace the synthetic version of this carotenoid that is generally used to colour salmonids and shrimp^47^. Precisely these nutritional characteristics make *Tigriopus* biomass a potential substitute for Antarctic krill and Calanus oils for human consumption as a nutraceutical. Other researchers and ourselves believe that capturing Antarctic krill and *Calanus finmarchicus* in the ocean to supply this market is detrimental to the ecosystem^48,49^. Therefore, land-based production of these nutrients can help reduce these fishing practices.

*Tigriopus* is an important model organism for research on ecotoxicology^50^, physiology^51^, genetics^52^ and genomics^53,54^. At the scale described, the bioreactor would produce *Tigriopus* in sufficient quantities to investigate in these fields. Nevertheless, for mass production of zooplankton biomass, cost-effective and environmentally friendly upscaling must be achieved. Upscaling will almost certainly involve adopting artificial lighting with submersible lights^55^. Both phases of the bioreactor should be illuminated, since the microalgae need light also in this phase, and *Tigriopus* has a complex phototactic behavior that needs to be met^56,57^. An automatic zooplankton harvesting system is also necessary, a topic we are working on in our group. Furthermore, nutrient supplementation must be carefully planned and monitored. For example, organic sources of nitrate and phosphate should be used instead of their sodium or potassium salts to avoid accumulating these cations in the bioreactor.

Recently, it has been proposed that an invertebrate aquaculture system could be a good option to produce food on long-term space travel^58^. As the authors suggest, such a system should be a closed circuit, produce food for the invertebrates on-site and facilitate the maintenance of water quality. Our process meets these requirements, making it an option to consider for this exciting application. However, astaxanthin is not an essential carotenoid for humans. On the other hand, β-carotene and lutein are essential in human physiology, and have been shown to protect DNA from radiation damage^62^. We have tested our system with *Dunaliella tertiolecta* and *Artemia franciscana*, and we have produced *Artemia* enriched in these two carotenoids.

## Conclusion

The work described here suggests that closed-loop ‘green water’ polycultures can help cross one of the biotechnological frontiers humans are still facing: The mass production of zooplankton. However, we must first consider certain limitations of the trial described here, which must be addressed in future developments. We have not evaluated the rate of formation and remineralisation of sediments produced by faecal pellets, carapaces, and decaying algae and bacteria. We have also not measured dissolved oxygen and ammonia concentrations. Although these factors did not appear to affect the growth of *Tetraselmis* or *Tigriopus*, the continuous evaluation of these parameters will be essential in reactors in continuous operation. To address these limitations and move towards large-scale production, as a next step, we propose to investigate the behaviour of the resident species in a water column with a depth that reflects commercial scale. If such a scale is achieved, our process can contribute to the clean development of aquaculture and food security. The deployment of this technology would reduce overfishing for aquaculture and land clearing for crops, this helping humanity to decouple its survival from the exploitation of the environment.

## Material and methods

### Reagents

Sodium nitrate (plant and cell-culture tested), sodium molybdate dihydrate, copper (II) sulfate pentahydrate, cobalt (II) chloride hexahydrate, butylated hydroxytoluene (BHT) and triethylamine were purchased to Sigma-Aldrich (Arklow, Ireland). Iron (III) chloride hexahydrate, zinc sulphate heptahydrate, manganese (II) chloride tetrahydrate, potassium dihydrogen phosphate and HPLC grade methanol and ethanol 96% were purchased to VWR International Ltd (Dublin, Ireland). Thiamine HCl (vit*.* B1), biotin (vit*.* H), cyanocobalamin (vit*.* B12) and Supelco 37 Component FAME Mix were sourced from Sigma-Aldrich. Tropic Marine Pro-Reef marine salt (Tropic Marin, Wartenberg, Germany) were obtained from a local retailer (McGuire’s Garden Centre, Woodstown, Ireland). Carotenoid standards were obtained from CaroteNature GmbH (Müsingen, Switzerland). The Pierce BCA protein assay kit was obtained from Thermo Fisher Scientific (Dublin, Ireland). The commercial krill and Calanus oil supplements Prowise SUPERBA*krill* Oil, Lot 1074600100 (Prowise Healthcare Ltd, London, UK) and Arctic Ruby (Calanus) Oil, Lot 1702071 (Calanus AS, Tromsø, Norway) were purchased online. HPLC grade methyl tert-butyl ether (MTBE) was supplied by Fisher Scientific (Dublin, Ireland).

### Bioreactor parts

Acrylic tubes and panes were supplied by Alternative Plastics Ltd (Nuneaton, UK). UPVC overflow pipes (Sanbra Fyffe Ltd, Dublin) were purchased in a local retailer (Morris's DIY and Builders Providers, Waterford, Ireland). Cut of acrylic parts was performed locally, at Q-Signs (Waterford). The black PVC water trough (Model DT1000FF, 2180 × 1180 × 840mm, JFC Agri, Tuam, Ireland, with 1000 L of capacity) was purchased to Glanbia CountryLife (Kilmeaden, Ireland). The TMC REEF-Pump 12000 DC water pump (Tropical Marice Centre Ltd, Chorleywood, UK), the Bubble Magus DSP4000 pump skimmer pumps (Jiang Aquarium Equipment Co, Ltd, China), the Koi KA 50 air pump (Koi Company, Langenselbold, Germany), the XY-380 Oxygen Biochemical sponge filters, the Fluval G6, 75-micron mechanical pre-filter cartridges (Fluval, Castleford, UK), the ceramic rings (unglazed pieces of fired ceramic) and the aquarium heaters were obtained from McGuire’s Garden Centre (Woodstown, Ireland). Throughout the trial, the enriched Guillard’s seawater F2 medium^59^ (salinity 35 g L^−1^) was used, with KH_2_PO_4_ in substitution of NaH_2_PO_4_ as a source of phosphorus.

### Monitoring

Bioeactor operation was monitored using the AlgaeConnect System (Algae Lab Systems, Boulder, CO, USA), consisting of a HubBox ALS-ACH with a wireless hub ALS-WHUB1-24 and two wireless monitor and control boxes ALS-SPARC-2A. The sensors were two pH/temp sensors ALS-PHT1. Nitrate, phosphate and iron were measured using the fluorometric assay kits Visocolor ECO (Macherey-Nagel GmbH & Co. KG, Düren, Germany) and a spectrophotometer (NANOCOLOR Kompaktphotometer PF-12 Plus, Macherey-Nagel GmbH & Co KG, Dueren, Germany), sourced from Apex Scientific Ltd (Maynooth, Ireland). Specifically, nitrate was measured with Visocolor ECO Test 5-41, phosphate with Visocolor ECO Test 5-84, and Fe II and Fe III with Visocolor ECO test 5-26. Algae concentration was estimated measuring absorbance at 690 nm using the Kompaktphotometer PF-12 Plus and correlating with microalgae wet weight^60^. The formula obtained was y = 1.8372x + 0.0405, where *y* is the microalgae concentration (g L^−1^ wet weight) and *x* is the absorbance at 690 nm.

### Organisms

The microalgae species *Tetraselmis chui* Butcher (strain 8/6) was obtained from the Culture Collection of Algae and Protozoa SAMS Limited (Argyll, Scotland, UK). The zooplankton species *Tigriopus californicus* was obtained from Reefphyto Ltd (Newport, UK).

### Biomass characterization

For dry mass estimation, three samples of freshly harvested biomass were heated at 90 °C during 24 h in a Memmert UN30 Oven (VWR International Ltd, Dublin, Ireland). Biomass analysis was commissioned to ALS Global (Clonmel, Ireland). For protein estimation, we analysed *Tigriopus* biomass using the Bicinchoninic acid (BCA method), using SDS 2 % and Urea 8 M, as directed by the manufacturer instructions.

### Oil extraction

For oil extraction from *Tigriopus*, circa 30 g of frozen biomass was freeze-dried for 24 h in a Coolsafe Superior Touch 55-80 freeze dryer, (Lavogene Aps, Bjarkesvej, Denmark). The dry biomass was homogenized with 300 mL of ethanol 96% with 0.1% BHT per 100 g of sample in an Ultra-Turrax T50 basic homogenizer with an S 50 N–W 65 SK cutting head (IKA-Werke GmbH & Co. KG, Stafen, Germany). The resulting slurry was incubated in 250 mL polypropylene centrifuge bottles at 200 rpm and 35 °C. The bottles were centrifuged at 4248 g in a Sigma 3–18 K centrifuge for 10 min at room temperature to remove debris and denatured proteins. The biomass was re-suspended and extracted two more times with new ethanol 96% with 0.1% BHT, and the extractions were combined and evaporated in a rotary evaporator RII (Buchi, Mason Technology Ltd., Dublin, Ireland) at 35 °C. The oleoresin was re-suspended in hexane and washed with dichloromethane:methanol in a proportion of 2:1 (v/v) and aqueous sodium chloride 10% (w/v). The oil obtained was determined gravimetrically and related to the biomass extracted as percentage of dry weight.

### Carotenoid quantification

Carotenoids were quantified in a UV-vis spectrophotometer UVmini-1240 (Shimadsu) using the molar extinction coefficient for astaxanthin 125 × 10^3^ L mol^−1^ cm^−1^ in hexane^61^. Carotenoid content was referred to sample dry weight or oil weight*.*

For HPLC analysis, the oil was separated and quantified in an HPLC system 1200 Series (Agilent Technologies, USA), equipped with a Diode Array Detector, quaternary pump, degasser, thermostatically controlled column compartment and thermostatically controlled autosampler. The column was a C30 (250 × 4.6 mm i.d., 3 µm; YMC Europe, Dinslaken, Germany) with a guard column containing a guard cartridge with the same chemistry of the column (10 × 4 mm i.d., 3 µm). The flow rate was 1 mL min^−1^ with a linear gradient from 100% A (methanol:methyl tert-butyl ether:water:triethylamine (30:10:1:0.05, v/v/v/v)) to 20% B (methanol: methyl tert-butyl ether (1:1, v/v)) within 10 min, then to 100% B within 1 min, keeping this condition for another 24 min. The solvents were returned to the starting conditions within 1 min, and the column temperature was set at 25 °C.

### Fatty acid quantification

The fatty acids present in the oil extracted from *Tigriopus* were quantified by gas chromatography coupled to flame ionization detection using an Agilent Technologies7890B chromatograph (Agilent Technologies, California, USA) equipped with an FID detector and a Thermo TR-FAME 260M142P column (30 m length, 250 µm inner diameter, 0.25 µm film thickness). Nitrogen was used as carrier gas, with flow rate 1.5 ml min^−1^ and electronic pressure control at 20.8 psi. The temperature ramp was as follows: 140 °C for 1 minute followed by an increase of 10 °C min^−1^ until 210 °C for 8 minutes, and an increase of 2 °C min^−1^ until the final temperature of 230 °C, which was kept for 25 minutes. Post run temperature was 50 °C. Injection volume was 1 µL with split 1:10.

Fatty Acid Methyl Esters (FAME) were prepared as follows: 5 mg of oil samples were weighted in a 15 mL polypropylene tube. Then, 3 mL of methanolic sulphuric acid 2% v/v containing the internal standard (IS) methyl lignocerate at a final concentration of 0.1 mg/mL were added to each tube. The samples were vortexed until complete mixing of the oil. 0.5 mL of each sample were transferred to a Duran glass tube. The tubes were heated at 80 °C for 2 h, and the reaction was neutralized with aqueous NaCl 0.9%. 0.5 mL of hexane were added, the tubes were vortexed and left to settle to form two layers. The hexane layer was transferred to a GC vial for analysis. FAME were identified by comparison with Supelco 37 Component FAME Mix and Primrose and Hemp oil. A response factor for each analysis was calculated with the internal standard run independently in triplicate, and dividing the average area obtained by the amount of IS injected (0.01 ng).

### Statistical analysis

The fatty acid composition of Tigriopus oil (*n* = 3), Antarctic krill oil (*n* = 2) and Calanus oil (*n* = 2) was compared using Independent Samples 2-tailed t-tests with 95% significance confidence interval. The significance used depended on the homogeneity of the variances, as calculated with Levene’s test.
